# Involvement of mTOR pathway in neurodegeneration in NSF-related developmental and epileptic encephalopathy

**DOI:** 10.1093/hmg/ddad008

**Published:** 2023-01-16

**Authors:** Takahiro Hayashi, Naoko Yano, Kengo Kora, Atsushi Yokoyama, Kanako Maizuru, Taisei Kayaki, Kinuko Nishikawa, Mitsujiro Osawa, Akira Niwa, Toshiki Takenouchi, Atsushi Hijikata, Tsuyoshi Shirai, Hisato Suzuki, Kenjiro Kosaki, Megumu K Saito, Junko Takita, Takeshi Yoshida

**Affiliations:** Department of Pediatrics, Kyoto University Graduate School of Medicine, Kyoto 606-8507, Japan; Department of Pediatrics, Kyoto University Graduate School of Medicine, Kyoto 606-8507, Japan; Department of Pediatrics, Kyoto University Graduate School of Medicine, Kyoto 606-8507, Japan; Department of Pediatrics, Kyoto University Graduate School of Medicine, Kyoto 606-8507, Japan; Department of Pediatrics, Kyoto University Graduate School of Medicine, Kyoto 606-8507, Japan; Department of Pediatrics, Kyoto University Graduate School of Medicine, Kyoto 606-8507, Japan; Department of Pediatrics, Kyoto University Graduate School of Medicine, Kyoto 606-8507, Japan; Thyas Co. Ltd, Kyoto 606-8501, Japan; Department of Clinical Application, Center for iPS Cell Research and Application (CiRA) Kyoto University, Kyoto 606-8507, Japan; Department of Clinical Application, Center for iPS Cell Research and Application (CiRA) Kyoto University, Kyoto 606-8507, Japan; Department of Pediatrics, Keio University School of Medicine, Shinjuku, Tokyo 160-8582, Japan; Faculty of Bioscience, Nagahama Institute of Bio-Science and Technology, Nagahama, Shiga 526-0829, Japan; School of Life Sciences, Tokyo University of Pharmacy and Life Sciences, Hachioji, Tokyo 192-0392, Japan; Faculty of Bioscience, Nagahama Institute of Bio-Science and Technology, Nagahama, Shiga 526-0829, Japan; Center for Medical Genetics, Keio University School of Medicine, Shinjuku, Tokyo 160-8582, Japan; Center for Medical Genetics, Keio University School of Medicine, Shinjuku, Tokyo 160-8582, Japan; Department of Clinical Application, Center for iPS Cell Research and Application (CiRA) Kyoto University, Kyoto 606-8507, Japan; Department of Pediatrics, Kyoto University Graduate School of Medicine, Kyoto 606-8507, Japan; Department of Pediatrics, Kyoto University Graduate School of Medicine, Kyoto 606-8507, Japan

## Abstract

Membrane fusion is mediated by soluble *N*-ethylmaleimide-sensitive factor attachment protein receptor (SNARE) proteins. During neurotransmitter exocytosis, SNARE proteins on a synaptic vesicle and the target membrane form a complex, resulting in neurotransmitter release. *N*-ethylmaleimide-sensitive factor (NSF), a homohexameric ATPase, disassembles the complex, allowing individual SNARE proteins to be recycled. Recently, the association between pathogenic *NSF* variants and developmental and epileptic encephalopathy (DEE) was reported; however, the molecular pathomechanism of NSF-related DEE remains unclear. Here, three patients with *de novo* heterozygous *NSF* variants were presented, of which two were associated with DEE and one with a very mild phenotype. One of the DEE patients also had hypocalcemia from parathyroid hormone deficiency and neuromuscular junction impairment. Using PC12 cells, a neurosecretion model, we show that NSF with DEE-associated variants impaired the recycling of vesicular membrane proteins and vesicle enlargement in response to exocytotic stimulation. In addition, DEE-associated variants caused neurodegenerative change and defective autophagy through overactivation of the mammalian/mechanistic target of rapamycin (mTOR) pathway. Treatment with rapamycin, an mTOR inhibitor or overexpression of wild-type NSF ameliorated these phenotypes. Furthermore, neurons differentiated from patient-derived induced pluripotent stem cells showed neurite degeneration, which was also alleviated by rapamycin treatment or gene correction using genome editing. Protein structure analysis of NSF revealed that DEE-associated variants might disrupt the transmission of the conformational change of NSF monomers and consequently halt the rotation of ATP hydrolysis, indicating a dominant negative mechanism. In conclusion, this study elucidates the pathomechanism underlying NSF-related DEE and identifies a potential therapeutic approach.

## Graphical abstract

**Figure:**
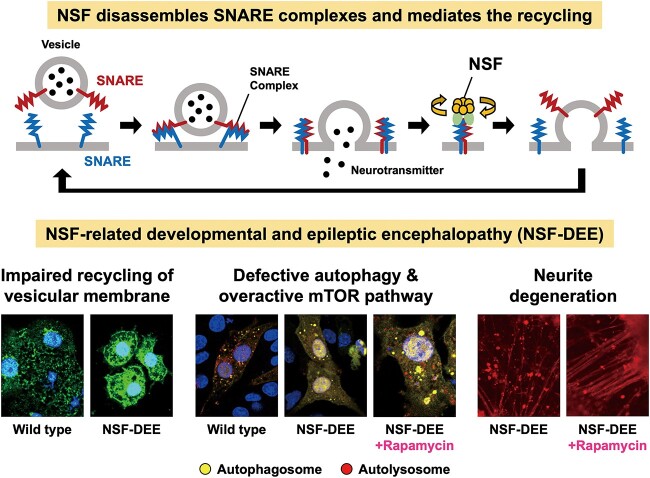

## Introduction

Membrane fusion is one of the most fundamental intracellular events and is essential to many biological processes, including membrane trafficking, neurotransmitter exocytosis and hormone secretion, in all living organisms. Membrane fusion is mediated by a set of proteins known as soluble *N*-ethylmaleimide-sensitive factor attachment protein receptor (SNARE). In neurotransmitter exocytosis, three kinds of SNARE proteins (i.e. VAMP2 anchored on the synaptic vesicle membrane and syntaxin-1A and SNAP25 on the targeted presynaptic membrane) form a SNARE complex, resulting in membrane fusion and neurotransmitter release (1,2). *N*-ethylmaleimide-sensitive factor (NSF), a homohexameric ATPase, binds a SNARE complex via adaptor proteins known as αSNAP (NSF-attachment protein alpha) and disassembles the complex by utilizing energy from adenosine triphosphate (ATP) hydrolysis. Individual SNARE proteins will be recycled in other membrane fusion events after disassembly by NSF (3–5). Various specific SNARE proteins have been discovered in different organelles and tissues, although NSF is the only protein that dissociates SNARE complexes in most eukaryotic organisms (6). NSF, therefore, plays an irreplaceable role in the recycling of SNARE proteins, and yet the relationship between NSF and human diseases remains unclear.

Developmental and epileptic encephalopathy (DEE) is a devastating condition characterized by two aspects: highly active epilepsy and delayed development (7). In many cases of DEE, delayed development is the result of both intractable epilepsy and neurodegeneration induced by genetic abnormality. Over 100 genes have been identified to cause DEE, some of which are associated with SNARE-mediated membrane fusion. For example, syntaxin-binding protein 1, a chaperone protein for syntaxin-1A, is responsible for DEE4, also known as Ohtahara syndrome (8). Ohtahara syndrome is characterized by very early onset severe epilepsy, a burst-suppression electroencephalogram (EEG) pattern and profoundly delayed development. Pathogenic *de novo* variants in SNARE proteins (e.g. SNAP25 and VAMP2) also cause neurodevelopmental diseases (9,10). These conditions, because of the mutation in proteins associated with SNARE-mediated membrane fusion, were recently grouped as ‘SNAREopathies.’ We previously reported two unrelated patients with pathogenic de novo variants in NSF (P563L and A459T) who showed a burst-suppression EEG pattern from birth and profound developmental delay (11). We demonstrated that GAL4-driven expression of the mutant NSF severely affected eye development in *Drosophila*, suggesting a dominant negative mechanism. However, the molecular pathomechanism of NSF-related DEE has not been elucidated, and no specific treatment is available, as in the vast majority of other DEEs.

This study aimed to reveal the pathomechanism of NSF-related DEE and determine treatment target pathways. Three patients with *de novo* heterozygous *NSF* variants, P563L, A459T and E715K (two DEE-related variants and a newly identified variant associated with a very mild phenotype), were presented. The ability of variant-carrying NSF to govern intracellular membrane fusion processes was analyzed using PC12 cells, which are commonly used as a neurosecretion model. Neurons differentiated from induced pluripotent stem (iPS) cells derived from one patient were also analyzed. Furthermore, the impact of the variants on the hydrolysis function of NSF on the basis of protein structural analysis was evaluated. We hypothesized that dysfunctional NSF would hinder the vesicular recycling process, resulting in aberrant activities associated with intracellular membrane fusion because of a deficiency of vesicular proteins.

**Table 1:** Summary of the clinical findings and genetic analyses of the three patients

**Characteristic**	**Patient 1**	**Patient 2**	**Patient 3**
Sex	Female	Female	Female
Age at study, years	6 years	Early infancy (deceased)	4 years
*NSF* variant (GRCh37) (NM_006178.4)	17:44791279C > T c.1688C > T; p.(P563L)	17:44782125G > A c.1375G > A; p.(A459T)	17:44828968G > A c.2143G > A; p.(E715K)
Inheritance	*De novo*	*De novo*	*De novo*
Age at seizure onset	Neonatal period	At birth	No seizure
Epilepsy	Myoclonic epilepsy	Myoclonic epilepsy	Not applicable
EEG	Burst-suppression	Burst-suppression	Normal
Development	No psychomotor milestones	Unknown	Normal
Other features	No voluntary respiration from birth, anemia, hypoparathyroidism, hypocalcemia, impaired neuromuscular junction	Vomiting at birth	Mild spastic paraplegia and startle response (both only in infancy)

## Results

### Clinical findings and genetic analysis

Patients 1 and 2 were previously reported to have been diagnosed with DEE related to heterozygotic *NSF* variants (11). Patient 1 carrying the P563L variant of *NSF* was born at 33 weeks of gestation to nonconsanguineous Japanese parents. From her birth, she had no spontaneous respiration and was completely dependent on a respirator. She began experiencing frequent myoclonic seizures as a neonate. Her EEG showed a continuous burst-suppression pattern. The brain magnetic resonance imaging (MRI) did not reveal any structural abnormalities or destructive lesions in early infancy. The subsequent MRIs showed progressive atrophy of the cerebral cortex, brainstem and cerebellum, as well as delayed myelination (Supplementary Material, Fig. S1A–C). She required regular red blood cell transfusions during the fetal period and up to age 6 months because of severe anemia of unclear etiology. After recovery from transfusion dependence, she has continuously exhibited macrocytic anemia and anisocytosis (Supplementary Material, Fig. S1D). At age six, she had not achieved any developmental milestones, and she persistently showed refractory seizures and a burst-suppression EEG pattern (Supplementary Material, Fig. S1E).

In addition, she required calcium supplementation because of severe hypocalcemia, and the blood level of intact parathyroid hormone (PTH) was always below the lower limit. PTH is known to be stored in vesicles within parathyroid cells and released by exocytosis-like neurotransmitters. Except for PTH, a thorough endocrinological examination found no abnormalities. Examination of the function of acetylcholine exocytosis at neuromuscular junctions demonstrated the decremental pattern of muscle contractions following repetitive nerve stimulation (Supplementary Material, Fig. S1F). In contrast, degranulation (cytotoxic granule exocytosis) assay of CD8+ T cells was normal, and the patient was immunocompetent. These findings suggested that the patient’s exocytosis response was impaired in conditions with fast repetition as opposed to conditions with a single shot.

Patient 2 carrying the A459T variant of *NSF* was born at 37 weeks of gestation to nonconsanguineous Japanese parents. She began vomiting and had tonic seizures immediately after birth. On the 9th day after birth, EEG revealed a burst-suppression pattern, independent of the sleep–wake state. She did not have hypocalcemia, in contrast to Patient 1. She died of respiratory failure in early infancy. Patient 3 presented mild spastic paraplegia in infancy and a startle response to photic stimulation, but electroencephalography was normal. A *de novo* missense variant, c.2143G > A; p. (E715K), in *NSF* was identified after she was enrolled in a Japanese national project known as the Initiative on Rare and Undiagnosed Disease (12). At age 4 years, the patient developed normally and had no symptoms. We, therefore, considered that the E715K variant can be very mildly damaging.

Table 1 displays a summary of the clinical findings and variant features of the three patients. The candidate gene variants identified by trio-based whole exome sequencing in Patients 1 and 2 are listed in Supplementary Material, Table S1. There were no additional gene variants that can be responsible for anemia or respiratory failure.

### DEE-associated *NSF* variants impaired the recycling of vesicular membrane proteins

**Figure 1:**
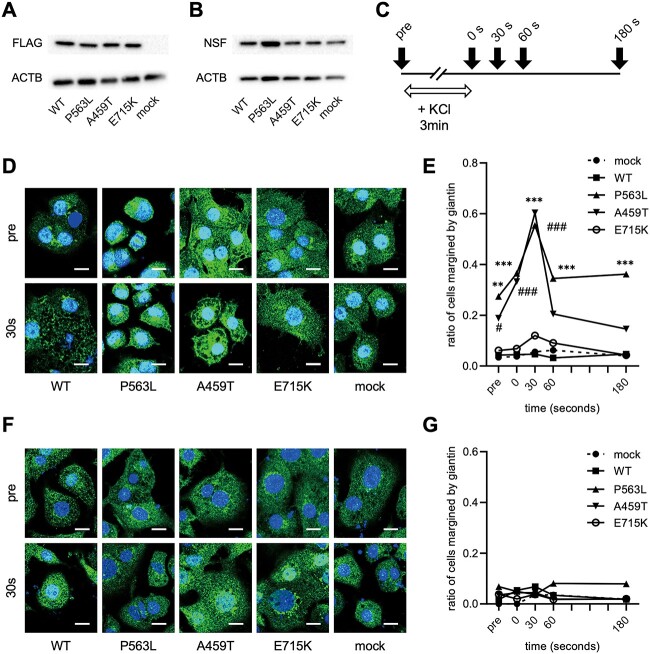
Disturbed recycling of vesicular membrane protein in NSF with DEE-associated variants. Representative images of western blot analysis for FLAG (**A**) and NSF (**B**) in PC12 cells that stably express WT or variant-carrying NSF tagged with FLAG. Beta-actin (ACTB) is used as a loading control. (**C**) Time course of immunofluorescence assay for giantin. The white arrow represents the duration of KCl stimulation. The black arrows represent each time point of fixation for immunofluorescence. (**D**) Confocal microscopic images of immunofluorescence assay for giantin (green) in each PC12 cell line. These images are obtained at baseline and 30 s after 80 mm KCl stimulation. The nuclei are stained with DAPI. Scale bar = 10 μm. (**E**) Ratio of giantin-margined cells in each PC12 cell line. (**F**) Ratio of giantin-margined cells in each PC12 cell line with transient expression of NSF^WT^. (E) and (F) The data represent the ratio of 35–70 cells. Statistical difference is evaluated using ^*^P < 0.05, ^*^^*^P < 0.0 chi-squared test and Fischer’s exact test.1, ^*^^*^^*^P < 0.001 P563L versus WT, ^#^*P* < 0.05, ^###^*P* < 0.001 A459T versus WT.

To study the vesicular recycling process, we utilized PC12 cells derived from the adrenal glands of rats, a widely employed model for neurosecretion (13). PC12 cells stably expressing FLAG-tagged NSF were generated, and the results confirmed that each cell line expressed FLAG and NSF at comparable levels (Fig. 1A and B). In PC12 cells, vesicle exocytosis is activated in response to KCl stimulation (14), thus, the distribution of vesicular proteins was evaluated using immunofluorescence assays at various time points before and following KCl stimulation (Fig. 1C).

Giantin (a transmembrane protein in Golgi and vesicles) was mostly localized around the nucleus at baseline and broadly dispersed in the cytoplasm 30 s after KCl stimulation in PC12 cells transfected with an empty vector (designated as mock), cells expressing wild-type (WT) NSF (NSF^WT^), and cells expressing NSF with E715K variant (NSF^E715K^) (Fig. 1D). In contrast, in PC12 cells expressing NSF with DEE-associated variants (NSF^DEE^), giantin was collected at the cell surface, and the proportion of giantin-margined cells increased and peaked 30 s after KCl stimulation (Fig. 1D and E). This giantin accumulation at the cell surface was completely improved by transient overexpression of NSF^WT^ (Supplementary Material, Fig. S2A; Fig. 1F and G). These findings revealed that DEE-associated *NSF* variants impaired recycling of vesicular membrane proteins.

### DEE-associated *NSF* variants inhibited vesicle enlargement following stimulation of exocytosis

**Figure 2:**
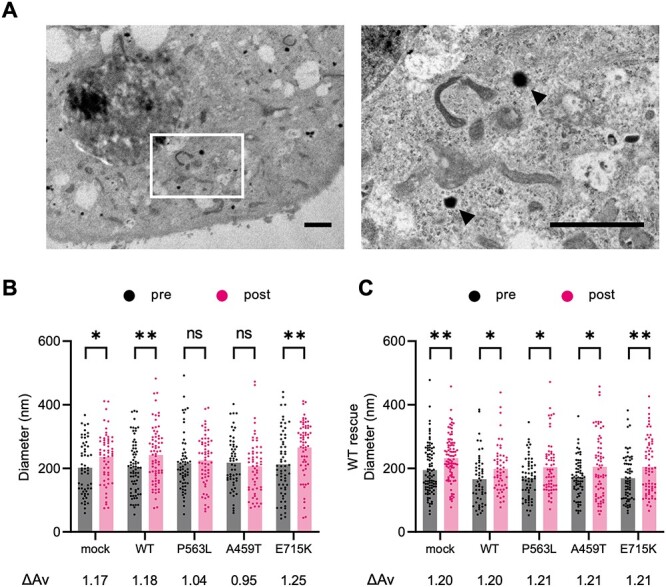
Impaired enlargement of vesicle diameter responding to stimulation of vesicle release in NSF with DEE-associated variants. (**A**) Representative electron microscopic images of a PC12 cell line. The arrowheads indicate LDCVs. Scale bar = 2 μm. (**B**) The mean diameter of LDCV before exocytotic stimulation (pre) and after exocytotic stimulation (post) in each PC12 cell line that stably expresses WT or variant-carrying NSF. (**C**) The mean diameter of LDCV in each PC12 cell line rescued by transient expression with NSF^WT^. Each dot represents the diameter of a vesicle. ΔAv means the ratio of averages of LDCV diameter after KCl stimulation to baseline. (B) and (C) The data represent the mean of 50–90 vesicles. Statistical difference is evaluated using paired *t*-test. ^*^P < 0.05, ^*^^*^P < 0.01.

PC12 cells possess catecholamine-containing vesicles called large dense-core vesicles (LDCVs) and release them in response to KCl stimulation (15–17). To further investigate the impact of impaired recycling of vesicular membrane proteins, we analyzed the size of LDCVs using electron microscopy (Fig. 2A). Following KCl stimulation, the LDCV size increased in mock, cells expressing NSF^WT^ and cells expressing NSF^E715K^ (Fig. 2B). Meanwhile, no increase in LDCV size was found in cells expressing NSF^DEE^. NSF^WT^ overexpression rescued the defective enlargement of LDCVs in cells expressing NSF^DEE^ (Fig. 2C). This indicated that the impaired recycling caused a shortage of vesicular membrane proteins and defective vesicle enlargement during continuous exocytosis. Collectively, the disruption of the vesicle exocytosis process by DEE-associated *NSF* variants is consistent with the patients’ symptoms, which include severely aberrant neuronal networks, diminished conduction at the neuromuscular junction, and defects in the release of intact PTH.

### Defective autophagy in PC12 cells expressing NSF with DEE-associated variants

**Figure 3:**
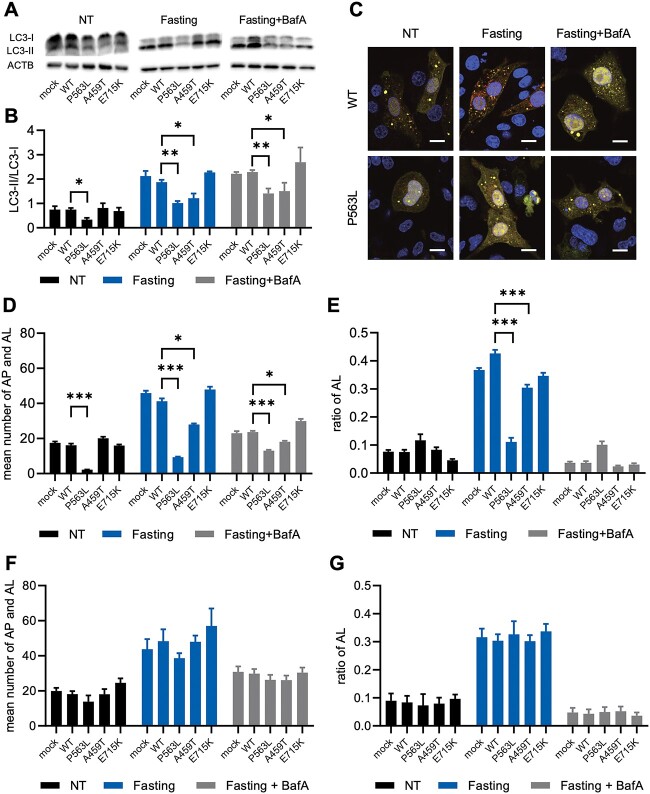
Impaired formation of AP and AL in NSF with DEE-associated variants. Representative images of western blot analysis for LC3 (**A**) and quantitative densitometric analysis of western blots (**B**) in PC12 cell lines that stably express WT or variant-carrying NSF. ACTB is used as a loading control. The data represent the mean ± standard error of the mean (SEM) of three independent experiments. (**C**) Representative confocal microscopic images of PC12 cell lines expressing NSF^WT^ or NSF^P563L^ transfected with tandem-fluorescent LC3 (tfLC3). The yellow puncta indicates AP, and the red puncta indicates AL. Scale bar = 10 μm. The mean number of total puncta (AP and AL) (**D**) and ratio of red puncta (AL) to total puncta (**E**) in tfLC3-transfected PC12 cell lines. The mean number of total puncta (**F**) and ratio of red puncta to total puncta (**G**) in tfLC3-transfected PC12 cell lines with NSF^WT^ overexpression. (D)–(G) The data represent the mean ± SEM of 10 cells. Statistical difference is evaluated using Welch’s *t*-test (B) and (D) and Fischer’s exact test (E). ^*^P < 0.05, ^*^^*^P < 0.01, ^*^^*^^*^P < 0.001.

In addition to intractable epilepsy, the pathomechanism of DEE is also related to neurodegeneration (8,18–20). Autophagy plays a significant part in numerous neurodegenerative disorders (21). To assess the effect of autophagy on NSF-related DEE, the autophagosome (AP) marker LC3 was analyzed in PC12 cells stably expressing NSF using western blotting. In addition, the ratios of LC3-II (active form) to LC3-I (precursor form), which reflect the activation of autophagy, were evaluated. Cells expressing NSF with P563L variant (NSF^P563L^) had lower LC3-II/LC3-I ratios than cells expressing NSF^WT^ under both no treatment (NT) and fasting conditions. Cells expressing NSF with A459T variant (NSF^A459T^) had a lower ratio only under fasting conditions (Fig. 3A and B). Bafilomycin A1 (BafA), which was previously reported to irreversibly inhibit the fusion of AP to lysosomes and drive autophagic activation (22), did not raise the LC3-II/LC3-I ratios in cells expressing NSF^DEE^ to the same level as in cells expressing NSF^WT^. These data show that both the activation and flux of autophagy were impaired in cells expressing NSF^DEE^.

Autophagic activation was impaired regardless of the fasting duration (Supplementary Material, Fig. S2A and B). Autophagic flux was further analyzed using red fluorescent protein (RFP)-green fluorescent protein (GFP)-tfLC3 reporter. In tfLC3-transfected cells, AP and autolysosomes (AL) were observed as yellow and red fluorescence, respectively. Normally, fasting increases the number of AP and AL, as well as the ratio of AL. BafA reversed these effects in the current study (Fig. 3C–E; Supplementary Material, Fig. S3A). The numbers of AP and AL were constantly lower in cells expressing NSF^P563L^, but they were only lower during fasting conditions in cells expressing NSF^A459T^ (Fig. 3D). The ratios of AL were lower in both P563L and A459T during fasting conditions (Fig. 3E). NSF^WT^ overexpression almost completely rescued the defective autophagy in cells expressing NSF^DEE^ (Fig. 3F and G; Supplementary Material, Fig. S3B). These results supported prior conclusions that NSF functions in AP fusion with lysosomes (23,24). Collectively, DEE-associated *NSF* variants impaired autophagic activation and flux to lysosome.

**Figure 4:**
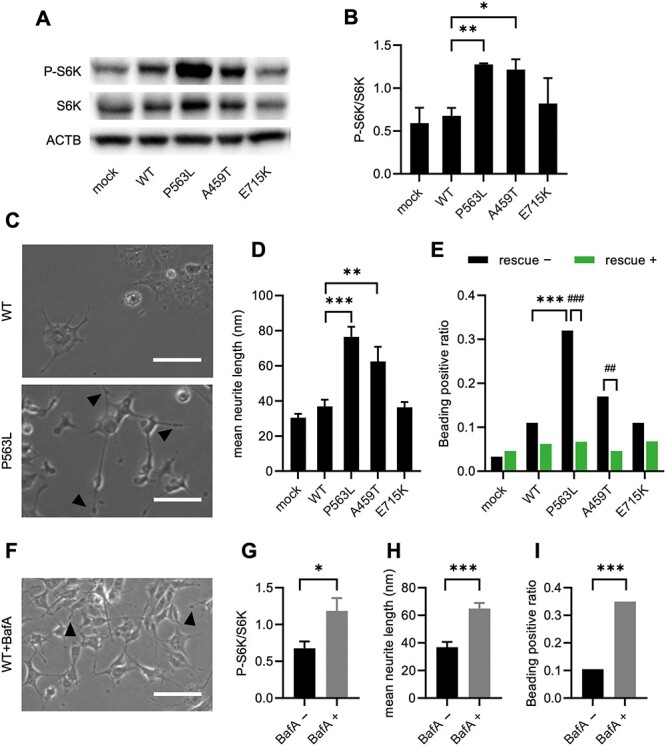
mTORC1 activation and neurite degeneration in NSF with DEE-associated variants. Representative images of western blot analysis for P-S6K and total S6K (**A**) and quantitative densitometric analysis of western blots (**B**) in each PC12 cell line. ACTB is used as a loading control. The data represent the mean ± SD of three independent experiments. (**C**) Representative phase contrast images of PC12 cell lines expressing NSF^WT^ or NSF^P563L^ after treatment with nerve growth factor. The arrow heads indicate axonal beading. Scale bar = 100 μm. (**D**) The mean neurite length in each PC12 cell line. The data represent the mean ± standard deviation of 40 neurites. (**E**) The ratio of beading-positive neurites to all neurites with rescue by transient expression with NSF^WT^ (*n* = 70–150 neurites). (**F**) A representative phase contrast image of PC12 cells stably expressing NSF^WT^ treated with 80 nm BafA. Scale bar = 100 μm. (**G**) Quantitative densitometric analysis of western blots for P-S6K and total S6K in PC12 cells stably expressing NSF^WT^ treated with BafA (*n* = 3 independent experiments). (**H**) The mean neurite length in BafA-treated PC12 cells stably expressing NSF^WT^ (*n* = 40 neurites). (**I**) The ratio of beading-positive neurites to all neurites in PC12 cell stably expressing NSF^WT^ treated with BafA (*n* = 80–110 neurites). Statistical difference is evaluated using Welch’s *t*-test (B, D, G and H) and Fischer’s exact test (E and I) ^*^P < 0.05, ^*^^*^P < 0.01, ^*^^*^^*^P < 0.001 versus WT. ^##^*P* < 0.01, ^###^*P* < 0.001 versus without rescue.

### DEE-associated *NSF* variants activated the mTORC1 pathway, leading to neurite degeneration

Autophagy is negatively regulated by the mammalian/mechanistic target of rapamycin complex 1 (mTORC1) pathway (25,26). It is known that excessive activation of the mTORC1 pathway causes neurodegeneration (27). To explore whether DEE-associated *NSF* variants influenced mTORC1 activity, we next analyzed the levels of phosphorylated S6K (P-S6K), a downstream target of the mTORC1, in PC12 cells stably expressing NSF using western blotting. The P-S6K/S6K ratios were elevated in cells expressing NSF^DEE^ (Fig. 4A and B). Examination of neurite shape in PC12 cells treated with nerve growth factor showed greater neurite length in DEE-associated variants than in WT, consistent with a hyperactive mTORC1 pathway (Fig. 4C and D). In addition, the neurites of cells expressing NSF^DEE^ had beading, an indication of neurite degeneration (Fig. 4C). Under NSF^WT^ overexpression, these beaded neurites by NSF^DEE^ were decreased to levels comparable to NSF^WT^ (Fig. 4E). To clarify the relationship among autophagy, the mTORC1 pathway and neurite degeneration, cells expressing NSF^WT^ were treated with BafA, which irreversibly blocked the fusion of AP with lysosomes. BafA treatment activated the mTORC1 pathway, elongated neurites and increased the fraction of neurites with beading (Fig. 4F–I). These results suggested that the impaired fusion of AP with lysosomes was associated with activation of the mammalian/mechanistic target of rapamycin (mTOR) pathway and was sufficient for neurite degeneration. Consequently, an activated mTORC1 pathway plays a key role in neurite degeneration induced by DEE-associated *NSF* variants.

### Rapamycin treatment ameliorated neurite degeneration in DEE-associated *NSF* variants

**Figure 5:**
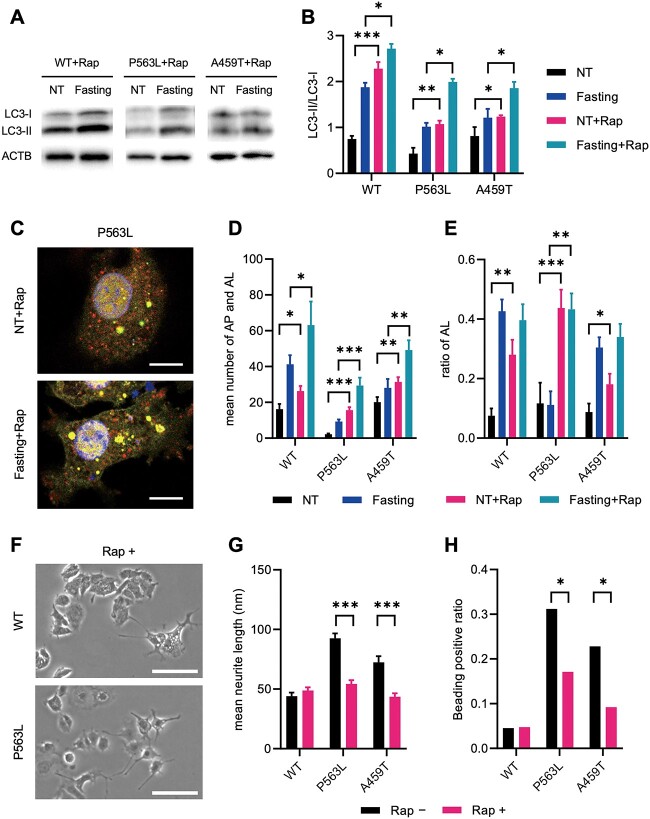
Rapamycin rescues the impaired autophagy and neurite degeneration in NSF with DEE-associated variants. (**A**) Representative images of western blot analysis for LC3 under treatment with rapamycin in PC12 cell lines that stably express NSF^WT^ or NSF^P563L^. ACTB is used as a loading control. (**B**) Quantitative densitometric analysis of western blots. The data represent the mean ± standard deviation of three independent experiments. (**C**) Representative confocal microscopic images of PC12 cells expressing NSF^P563L^ transfected with tandem-fluorescent LC3 (tfLC3) under treatment with rapamycin. The yellow puncta indicates AP, and the red puncta indicates AL. Scale bar = 10 μm. The (**D**) mean number of AP and AL and the (**E**) mean ratio of AL in each PC12 cell line transfected with tfLC3 (*n* = 10 cells). (**F**) Representative phase contrast images of PC12 cells expressing NSF^WT^ or NSF^P563L^ cultured with 200 nm rapamycin. Scale bar = 100 μm. (**G**) The mean neurite length in PC12 cells expressing NSF^WT^ or NSF^P563L^ (*n* = 40 neurites). (**H**) The ratio of beading-positive neurites to all neurites (*n* = 70–150 neurites). Statistical difference is evaluated using Welch’s *t*-test (B, D, E and G) and Fischer’s exact test (H). ^*^P < 0.05, ^*^^*^P < 0.01, ^*^^*^^*^P < 0.001.

Rapamycin suppresses mTORC1 activity, and this in turn activates autophagy; hence, we expect that rapamycin might improve the neurodegeneration in NSF-related DEE. Treatment with rapamycin increased the ratios of LC3-II to LC3-I in both PC12 cells expressing NSF^WT^ and those expressing NSF^DEE^ (Fig. 5A and B). Monitoring of autophagic flux with tfLC3 reporter system demonstrated that rapamycin increased the number of AP and AL, as well as the proportion of AL, in NSF^DEE^-expressing cells (Fig. 5C–E). These results indicated that rapamycin repaired the defective autophagic activation and AP-lysosome fusion. A recent research revealed that mTORC1 inhibits AP-lysosome fusion by phosphorylating VAMP8, a component of the SNARE complex required for AP-lysosome fusion (28). Moreover, rapamycin treatment shortened the neurites and reduced the fractions of beading-positive neurites in NSF^DEE^-expressing cells (Fig. 5F–H). Collectively, rapamycin ameliorated neurite degeneration caused by DEE-associated variants by inhibiting mTORC1 activity and enhancing autophagic flux. Interestingly, rapamycin also improved giantin accumulation at the cell surface in NSF^DEE^-expressing cells after stimulation of vesicle exocytosis (Supplementary Material, Fig. S4A and B). This finding suggested that rapamycin had a beneficial effect on the recycling of vesicular membrane proteins, as well as on neurodegeneration, in NSF-related DEE.

### Neurons differentiated from iPS cells exhibited mTORC1 activation and neurite degeneration

**Figure 6:**
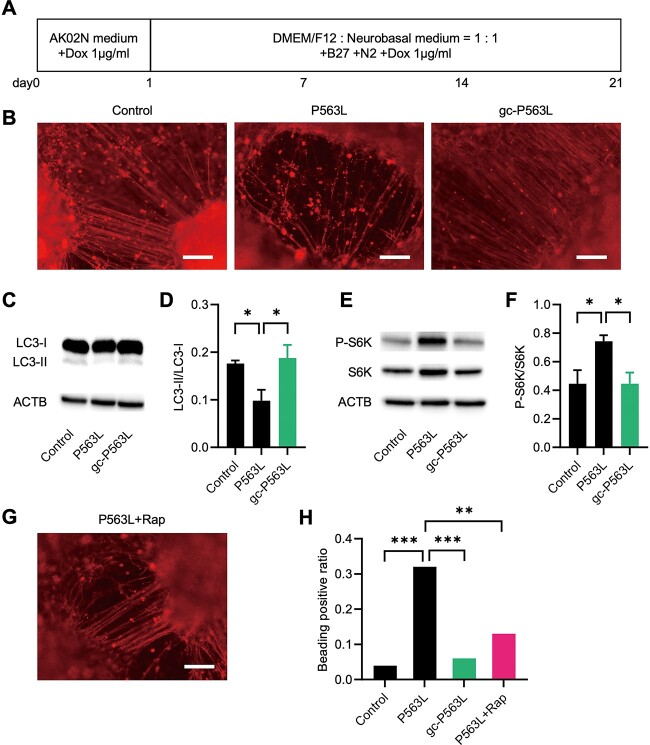
Gene correction using genome editing or treatment with rapamycin rescues the impaired autophagy and neurite degeneration in neurons differentiated from P563L iPS cells. (**A**) Time course of iPS cells for neurodifferentiation. (**B**) Representative microscopic images of immunofluorescence for TUBB3 (red) in neurons derived from control iPS cells (Control), P563L patient iPS cells (P563L) and gene-corrected P563L (gc-P563L). Scale bar = 100 μm. Representative images of western blot analysis for LC3 (**C**) and quantitative densitometric analysis of western blots of three independent experiments (**D**) in neurons derived from each iPS cell line. Representative images of western blot analysis for P-S6K and total S6K (**E**) and quantitative densitometric analysis of western blots of three independent experiments (**F**) in neurons derived from each iPS cell line. (C) and (E) ACTB is used as a loading control. (**G**) A representative microscopic image of immunofluorescence for TUBB3 (red) in neurons derived from P563L iPS cells cultured with 200 nm Rap. Scale bar = 100 μm. (**H**) The ratio of beading-positive neurites to all neurites in neurons derived from each iPS cell line (*n* = 200–280 neurites). Statistical difference is evaluated using Welch’s *t*-test (D and F) and Fischer’s exact test (H). ^*^P < 0.05, ^*^^*^P < 0.01, ^*^^*^^*^P < 0.001.

Fibroblasts were obtained from Patient 1 (P563L), and iPS cells were generated. The expression of pluripotent markers and the ability of iPS cells to differentiate into three lineages were validated (Supplementary Material, Fig. S5). Furthermore, using the CRISPR/Cas9 genome editing technique, a single amino acid substitution was introduced into the patient’s iPS cells, and gene-corrected clones (designated as gc-P563L), which served as a genetically identical control except for the *NSF* variant, were established. Then, control iPS cells derived from a healthy volunteer, P563L iPS cells and gc-P563L iPS cells were differentiated using Dox-inducible forced expression with NGN2, a regulator of neurogenic differentiation (Fig. 6A).

On day 21 of differentiation, neurons from each iPS cell line were fixed, and the neurites were examined for the neuronal marker TUBB3 via an immunofluorescence assay. P563L-neurons had sparse, curly and beaded neurites (Fig. 6B). As observed in PC12 cells, defective autophagy and activated mTORC1 pathway were observed in P563L-neurons (Fig. 6C–F). Gene correction rescued these findings. Next, the effect of rapamycin on neurons differentiated from the patient iPS cells was evaluated. Treatment with rapamycin restored the morphology of P563L-neurons and decreased the proportion of beading-positive neurites (Fig. 6G and H). In summary, P563L variant causes activation of the mTORC1 pathway and neurite degeneration in neurons differentiated from iPS cells, and this can be prevented by treatment with rapamycin.

### Deleterious effect of DEE-associated amino acid substitutions on hydrolysis function of NSF

**Figure 7:**
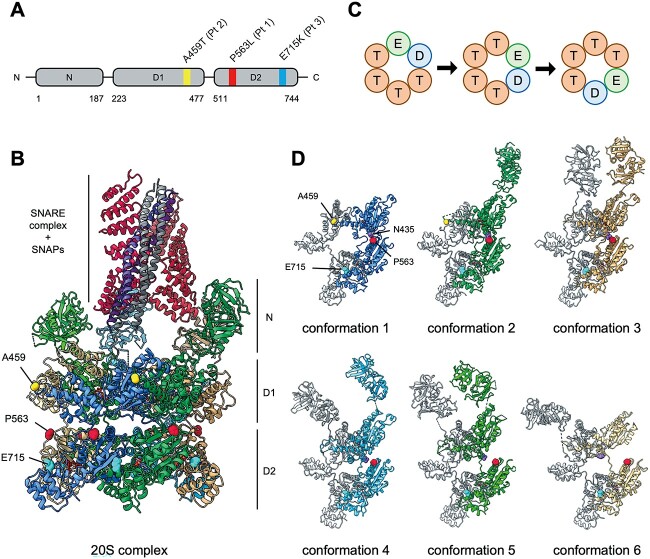
Predicted impact of amino acid substitutions on the protein structure and hydrolysis function of NSF. (**A**) Protein structural diagram of human NSF illustrating its domains and the variants identified in the patients (Pt). The numbers at the bottom indicate the number of amino acids. (**B**) The cryo-electron microscopy structure of complex 20S consists of a SNARE complex, SNAPs and NSF (Protein Data Bank code, 6MDN). Each protein is colored uniquely. The yellow, red and light blue sphere models correspond to amino acids A459, P563 and E715, respectively. The characters on the right denote the NSF domains. (**C**) The scheme of a rotary hydrolysis model. Each circle represents one individual monomer of NSF. The symbol in the circle represents each ATPase state as described further: E (circle in green), empty; D (circle in blue), ADP + Pi; T (circle in brown), ATP. ATP hydrolysis occurs sequentially. One monomer hydrolyzes ATP to ADP with conformational change, which is relayed to its adjacent monomer. The adjacent monomer will then hydrolyze its own ATP. (**D**) The conformational change of NSF. The purple sphere model corresponds to amino acid N435 within the D1 domain. The white structures indicate the conformation of an adjacent NSF. The molecular graphics images are prepared by using UCSF ChimeraX.

NSF protein comprises N, D1 and D2 domains (Fig. 7A). The D1 domain is principally responsible for ATPase activity, whereas the D2 domain is important for oligomerization. Homohexameric NSF proteins bind to the SNARE complex via adaptor proteins called soluble NSF-attachment proteins (SNAPs), forming a super-complex often referred to as a 20S complex (Fig. 7B). The A459T variant was positioned within a D1 domain, whereas the P563L and E715K variants were positioned within a D2 domain (Fig. 7A and B). Although the molecular mechanism of hydrolysis by hexameric NSFs remains unclear, recent studies support a rotary hydrolysis model (Fig. 7C) (29,30). This concept proposes that the hydrolysis of ATP proceeds sequentially. One monomer hydrolyzes ATP to adenosine diphosphate (ADP) with conformational change, which is relayed to its adjacent monomer. The adjacent monomer will then hydrolyze its own ATP (5,31,32). This rotation consequently generates the physical force necessary to dissociate a SNARE complex.

In this rotary model, conformational change of an NSF monomer is transmitted sequentially; hence, integration of a single aberrant NSF into the hexamer will halt the hydrolysis sequence (Fig. 7D). A459 is located at a helix–loop–helix motif that interacts with an adjacent monomer. A459 is modeled in conformation 1 and 2, but not in conformation 3–6. P563 is located at a site associated with interaction with the D1 and D2 domains. For example, the distance between N435 in the D1 domain and P563 in the D2 domain alters dynamically as conformational change progresses. In contrast, the position of E715 does not vary significantly between conformations. Collectively, it is suggested that the positions linked with DEE were pivotal for the transmission of conformational change during ATP hydrolysis. This hypothesis is consistent with the previous observation that the dominant negative pathogenic missense mutations show high odds at the molecular interfaces of homo-multimeric proteins (33).

## Discussion

The molecular pathomechanism of NSF-related DEE is yet to be clarified. This study assessed three *NSF* variants, of which two were associated with DEE (P563L and A459T) and one was associated with a very mild phenotype (E715K). The neurosecretion model demonstrated that DEE-associated variants impaired both recycling of vesicular membrane proteins and enlargement of vesicles in response to exocytotic stimulation. These results could be linked to an epileptic aspect of NSF-related DEE. Regarding the neurodegenerative aspect, we found that DEE-associated variants induced neurite degeneration through activation of the mTOR pathway and that rapamycin, an mTOR inhibitor, ameliorated the degeneration. Collectively, these results elucidate the pathomechanism underlying NSF-related DEE and identify a potential therapeutic approach. Through these analyses, we provide further support for the involvement of NSF in SNAREopathy and evidence that the mTOR pathway can be specifically targeted for the treatment of NSF-related DEE.

Although NSF is involved in various biophysiological processes, we initially focused on recycling SNARE proteins in exocytosis. NSF with DEE-associated variants led to impaired recycling of vesicular membrane proteins, which was exacerbated by exocytotic stimulation that increased the demand for SNARE proteins. These data support our hypothesis that rapid-cycling repetition of membrane fusion is specifically affected in patients with pathogenic *NSF* variants. In addition, vesicle enlargement in response to exocytotic stimulation was defective in PC12 cells with NSF^DEE^. This is likely because of the shortage of SNARE proteins required for membrane supply to expand vesicle size. Neurotransmitter exocytosis must be dynamically regulated in neural networks in response to changes in neuronal activity.

Therefore, it is not surprising that the inability of NSF to supply enough SNARE proteins caused epileptic encephalopathy. Similarly, defective vesicle exocytosis can explain a failure in PTH secretion and a decremental pattern of muscle contractions in repetitive stimulation of the neuromuscular junction observed in Patient 1; however, severe anemia cannot be explained. In a previous mouse study, knockdown of the erythroblast-specific SNARE proteins suppressed erythroblast proliferation (34). Pathogenic *NSF* variants may cause a shortage of erythroblast-specific SNARE proteins because of impaired recycling. We differentiated erythroblast progenitor cells from iPS cells of Patient 1, but did not detect any abnormalities in comparison to gene-corrected control cells (data not shown).

Defective autophagy is often associated with neurodegeneration (27). DMXL2 is a vesicular protein highly expressed at synaptic terminals, and its biallelic mutations cause DEE and impaired autophagy (20). We demonstrated that NSF^DEE^ also caused impaired autophagy. AP maturate by collecting membranes from various organelles and ultimately fuse with lysosomes (35). All these fusion processes are mediated by SNARE proteins. Recent research indicates that autophagic SNARE proteins are recycled rather than degraded in AL (36). NSF presumably plays a critical role in this recycling system. In addition, several studies have demonstrated that NSF is required for the fusion of the AP and lysosome (24,37,38). On the basis of the tfLC3 experiment in this study, NSF^DEE^-expressing PC12 cells had few AL in the fasting condition, similar to the BafA treatment condition. BafA commonly increases the proportion of LC3-II because of the reduction of LC3-II degradation in AL. However, in the current study, the proportion was decreased both in NSF^DEE^-expressing PC12 cells and in iPS-derived neurons with NSF^P563L^. Collectively, these findings indicated that DEE-associated *NSF* variants separately affected autophagic activation and AP–lysosome fusion.

Autophagy is negatively regulated by the mTOR pathway; nevertheless, the relationship between the two in pathological conditions is often complex. In this study, the mTOR pathway was activated in PC12 cells with NSF^DEE^, which further reduced autophagic activity. We also confirmed defective autophagy and an activated mTOR pathway in iPS cell-derived neurons in Patient 1. Treatment with the mTOR inhibitor rapamycin-activated autophagy and ameliorated neurodegeneration caused by DEE-associated *NSF* variants. These results indicated that the activated mTOR pathway is the primary contributor to NSF-related DEE and is responsible for defective autophagy and neurodegeneration. A previous report showed that trehalose, which activated autophagy in a manner distinct from mTOR inhibition, improved the motor and cognitive functions of mice with the *Lrrk2* variant that was characterized by NSF aggregation (39).

Rapamycin has been used clinically for several conditions, including the treatment of refractory epilepsy in patients with tuberous sclerosis complex (TSC) whose mTOR pathway is naturally activated (40). It has been reported that an mTOR inhibitor can be effective in the treatment for various epileptic conditions other than TSC (41–44). However, the specific mechanism behind the effectiveness of mTOR inhibitors against epilepsy remains uncertain. In this study, rapamycin also improved vesicular membrane recycling. Thus, rapamycin may have a favorable influence on vesicle exocytosis of neurotransmitters.

PC12 cells stably expressing NSF^DEE^ also produced endogenous NSF^WT^. Nevertheless, NSF functions were significantly damaged, and overexpression of NSF^WT^ restored them. These data show that the underlying mechanism may exhibit dominant negativity or haploinsufficiency. According to the gnomAD v2.1.1 population database of single nucleotide variant (accessed August 2022), the probability of being loss-of-function intolerant score of *NSF* was 0.018, below the cut-off value of 0.9 (45). This indicated that *NSF* cannot be classified as a haploinsufficient gene on the basis of genetic epidemiologic analysis. In addition, according to the copy-number variation database DECIPHER (accessed August 2022), none of the individuals with partial or complete deletion of *NSF* had epilepsy (46). These data suggested that haploinsufficiency was not the underlying mechanism of NSF-related DEE.

To further clarify the mechanism, we attempted to create NSF-knockout cells and compare the phenotype with other cell lines, but the experiments were unsuccessful, likely because NSF was essential for cell survival. It has been shown that homozygous NSF-knockout mice induce early embryonic lethality (47). Protein structure analysis of NSF showed that in contrast to the E715K variant, the DEE-associated P563L and A459T variants were located at positions that might disrupt the transmission of the conformational change of NSF monomers and consequently halt the rotation of ATP hydrolysis (29,32). Given that the amounts of an NSF carrying a pathogenic variant and a normal NSF are equal, the proportion of functional hexamers composed only of normal NSFs would be ⁓1.6% (0.5 raised to the sixth power). Heterozygous DEE-associated variants in *NSF* therefore affect nearly the whole function of NSF through a dominant negative mechanism. According to a previous study, E329Q variant located within the D1 domain of *NSF* had a dominant negative effect on mammalian cells (48). Other homohexameric ATPases, such as *VPS4A* and *AFG3L2*, have been identified to cause autosomal dominant diseases through a dominant negative mechanism (49,50). Collectively, these results indicate that the P563L and A459T variants in *NSF* most likely caused DEE via a dominant negative mechanism.

This study has some limitations. First, the number of patients was small. Unlike SNARE proteins, NSF is ubiquitously expressed and thus, NSF-related DEE may be distinguished from other SNAREopathies by extra-CNS symptoms. To clarify genotype–phenotype correlation, additional data on patients with *NSF* variants must be accumulated. Second, the pathogenicity of the E715K variant could not be established by this study. This might be because of the limitations of *in vitro* functional assays; however, the possibility that the variant is nonpathogenic was not ruled out. Third, the detailed mechanism of mTOR overactivation is unclear. Although it is assumed that dysfunctional NSF and BafA treatment have a common mechanism, further molecular investigation is required.

In conclusion, pathogenic variants in *NSF* disrupt several intracellular processes associated with membrane fusion through a dominant negative mechanism, including recycling of vesicular membranes, defective autophagy and overactivation of the mTOR pathway. Neurodegeneration mediated by the mTOR pathway is ameliorated with rapamycin treatment, indicating that rapamycin may serve as a targeted drug treatment for NSF-related DEE. Our study provided further evidence indicating the role of NSF in DEE or SNAREopathy.

## Materials and Methods

### Cell lines and cell culture

This study was approved by the Ethics Committee of Kyoto University Graduate School and Faculty of Medicine (approval number #R0091) and was conducted according to the guidelines of the Declaration of Helsinki. Informed consent was obtained from both the patients and their guardians.

PC12 cells were maintained in Dulbecco’s Modified Eagle’s Medium (DMEM) containing 10% heat-inactivated fetal calf serum supplemented with 50 μg/ml penicillin–streptomycin at 37°C and 5% CO_2_ conditions. For the observation of neurites, 50 ng/ml nerve growth factor (Nacalai Tesque, Kyoto, Japan; 24246-02) was added to the medium 48 h prior to the evaluation. Skin biopsy specimens were obtained from the P563L patient. The fibroblasts were expanded in DMEM containing 10% heat-inactivated fetal bovine serum and 50 μg/ml penicillin and streptomycin. iPS cells were generated as described previously (51). The control iPS cell line was kindly provided by Dr Takayuki Tanaka (Kyoto University). The pluripotency of control iPSC line was evaluated as previously described (52). Each iPSC line was cultured in Stem Fit AK02N medium (Takara Bio, Shiga, Japan; AJ100) on Matrigel (BD Biosciences, San Diego, CA, USA; 354230)-coated plate at 37°C and 5% CO_2_ conditions.

### Expression plasmid construction and DNA transfection

WT NSF was cloned from healthy control and inserted into p3 × FLAG-CMV14 vector (Sigma-Aldrich, Saint Louis, MO, USA) with a deoxyribonucleic acid (DNA) ligation kit (Takara Bio; 6023) using EcoRI and KpnI restriction sites following the manufacturer’s instructions. The complementary DNA (cDNA) encoding NSF with A459T or E715K variant was generated from WT NSF cDNA using a site-directed mutagenesis method. The NSF-p3 × FLAG-CMV14 vector was transfected into PC12 cells by electroporation using NEPA21 (NEPAGENE, Chiba, Japan). To screen the permanent expression clones, 0.7 mg/ml G418 (Nacalai Tesque) was used in the growth medium, and a single clone was isolated. For WT rescue experiments, WT-NSF-p3 × FLAG-CMV14 vector was transfected into each PC12 cell line by lipofection using Lipofectamine 2000 (Thermo Fisher Scientific, Waltham, MA, USA; 12566014) for transient expression.

### Immunofluorescence assay

Cells were cultured on coverglass chamber slides (IWAKI, Shizuoka, Japan; 5232-008), fixed with 4% paraformaldehyde for 10 min at 4°C, and washed three times with phosphate-buffered saline (PBS). The samples were then blocked with 3% bovine serum albumin (BSA) (w/v) in PBS-T (PBS with 0.05% (w/v) Triton X-100 solution) for 60 min at room temperature and then incubated with primary antibodies overnight at 4°C (diluted in PBS-T with 3% BSA). Thereafter, samples were washed three times with PBS and stained with secondary antibodies (1:500, Thermo Fisher Scientific; A11034 or A10037) for 60 min at room temperature. Nuclei were stained with 4′, 6′-diamidino-2-phenylindole (Nacalai Tesque; 19178-91). Images of the samples were acquired using a confocal microscope (Leica Camera AG, Wetzlar, Germany; TCS SP8) with a 633/1.40 oil-immersion objective lens (Zeiss, Oberkochen, Germany; Plan-Apochromatlan) and a camera (Zeiss; Axiocam HRm). After immunostaining for giantin, the number of giantin-margined cells, defined as cells in which giantin fluorescence intensity on the surface membrane exceeded 50% of giantin fluorescence in the surrounding nuclei, was counted.

### Transmission electron microscopy

PC12 cells were cultured on Matrigel-coated chamber slides (Thermo Fisher Scientific; 177445) and fixed overnight at 4°C with 3.6% paraformaldehyde and 2% glutaraldehyde and postfixed for 2 h with 1% osmium tetroxide in 0.1 m phosphate buffer. Following dehydration in a series of graded concentrations of ethanol, the samples were embedded in epoxy resin (Nacalai Tesque; 20829). Ultrathin sections (70 nm thickness) were prepared on an ultramicrotome (Leica Camera AG; EM UC6). Finally, the sections were inspected using an electron microscope (Hitachi, Tokyo, Japan; H-7650) after being stained with uranyl acetate and lead citrate. Transmission electron microscopy was performed at the Division of Electron Microscopic Study, Center for Anatomical Studies, Graduate School of Medicine, Kyoto University.

### Protein extraction and western blotting

Cultured cells were washed twice with PBS and lysed in M-PER buffer (Thermo Fisher Scientific; 78501) containing 2% (v/v) Protease Inhibitor Cocktail (Nacalai Tesque; 25955). Then, cell pellets were sonicated three times on ice. Protein concentrations of the lysates were measured using the Bio-Rad Protein Assay Kit (Bio-Rad, Hercules, CA, USA; 5000002) following the manufacturer’s instructions. The lysates were diluted with 5 × LiDS sample buffer and boiled for 5 min. The samples were separated on sodium dodecyl sulfate-polyacrylamide gels before being transferred onto polyvinylidene fluoride membranes (Millipore, Billerica, MA, USA; IPVH00010). The membranes were blocked for 1 h in 5% milk or 3% BSA in Tris-buffered saline (TBS) with 0.1% Triton X-100 (TBS-T) and incubated overnight at 4°C with a primary antibody (listed in Supplementary Material, Table S1). After three washes with TBS-T, the membranes were incubated for 60 min at room temperature with secondary antibodies. After three washes with TBS-T and two washes with TBS, Clarity Western ECL Substrate (Bio-Rad; 1705060) was used to identify the proteins. The bands were digitally detected using ChemiDoc XRS+ and quantified using Image Lab software (Bio-Rad).

### Analysis of autophagy

To analyze the activation of autophagy, we performed immunofluorescent analysis for LC3 in the following conditions: NT, fasting and fasting with treatment with BafA (Sigma-Aldrich; 1661). In the fasting condition, cells were cultured for 120 min in DMEM without amino acids (WAKO, Osaka, Japan; 048-33575). BafA (80 μm) was added to the medium. To suppress the mTOR activity, rapamycin (200 nm) was added to the medium. RFP-GFP-tandem fluorescent LC3 (tfLC3) reporter was used to monitor the flux of autophagy. Cells were transfected with tfLC3 plasmid (Addgene, Watertown, MA, USA; 21074) using Lipofectamine 2000 following the manufacturer’s instructions. After 48 h, the cells were fixed with 4% paraformaldehyde for 10 min at 4°C and washed three times with PBS. Images were acquired with a confocal microscope (Leica Camera AG; TCS SP8).

### Targeted gene correction in P563L-NSF iPSCs via CRISPR/Cas9 system

To establish a genetically identical control, CRISPR/Cas9 genome editing was utilized to introduce a single amino acid substitution into the patient iPS cells carrying the P563L variant in *NSF* (53). Using the online CRISPR design tool, CRISPRdirect (54), Cas9 target sites were determined as follows: 5′-TTTAGCTGCAAAAATTGCAGAGG-3′. Cas9 vector (Addgene; 62988) was digested using BbsI and ligated with a pair of the annealed oligos (20 bp target sequences) that were cloned from the guide RNA targeting the previously described sequence. Puromycin resistance cassette flanked by loxP sites was cloned from pENTER-DMD vector (Addgene; 60605). For construction of targeting vector, we used NEBuilder HiFi DNA Assembly Master Mix (New England BioLabs, Ipswich, MA, USA; E2621). All the vectors were sequenced to ensure that they contained the proper sequences. The Cas9 vector and targeting vector were introduced to the patient iPS cells by electroporation. After puromycin selection, iPS cells were transfected with Cre recombinase to eliminate the loxP-flanking puromycin resistance cassette.

### Neurogenic differentiation of iPS cells

Doxycycline (Dox)-inducible forced expression with *NGN2* was used to establish a robust and efficient neurogenic differentiation method from iPS cells, as previously described (55). Briefly, iPS cells were seeded on Matrigel-coated plates at a density of 2 × 10^5^ cells per well in a 24-well plate in a Stem Fit AK02N medium with 10 μm Y-27632 (Nacalai Tesque; 08945). After 24 h, Dox was added at 1 μg/ml. On day 2, the medium was replaced with 1:1 mixed Neurobasal medium (Thermo Fisher Scientific; 21103049) and DMEM/Ham’s F-12 (Sigma-Aldrich; 6421) containing 1 μg/ml Dox, 1 mm L-glutamine, B27 supplement (Thermo Fisher Scientific; 17504044) and N2-supplement (Thermo Fisher Scientific; 17502048). On day 21, protein was extracted for western blotting and immunostaining. Images were acquired with BZ-X (Keyence, Osaka, Japan).

### Protein structural analysis

To predict the effects of the variants on NSF, the three-dimensional structure of NSF with the variants was generated from the cryo-electron microscopy structure of 20S complex, which consisted of a SNARE complex from *Cricetulus griseus* and SNAPs and NSFs from *Rattus norvegicus* (Protein Data Bank ID, 6MDN) (32).

## Supplementary Material

Supplementary_Materials_R1_ddad008

## Data Availability

The data that support the findings of this study are available from the corresponding author, upon reasonable request.
