# Supplementary material for: Involvement of mTOR pathway in neurodegeneration in NSF-related developmental and epileptic encephalopathy
Source: Hum Mol Genet. 2023 Jan 16;32(10):1683–97. doi: 10.1093/hmg/ddad008 (PMC10162430; doi:10.1093/hmg/ddad008)
Supplement: Supplementary_Materials_R1_ddad008 [file supplementary_materials_r1_ddad008.zip › Supplementary_Materials_R1_ddad008.pdf]

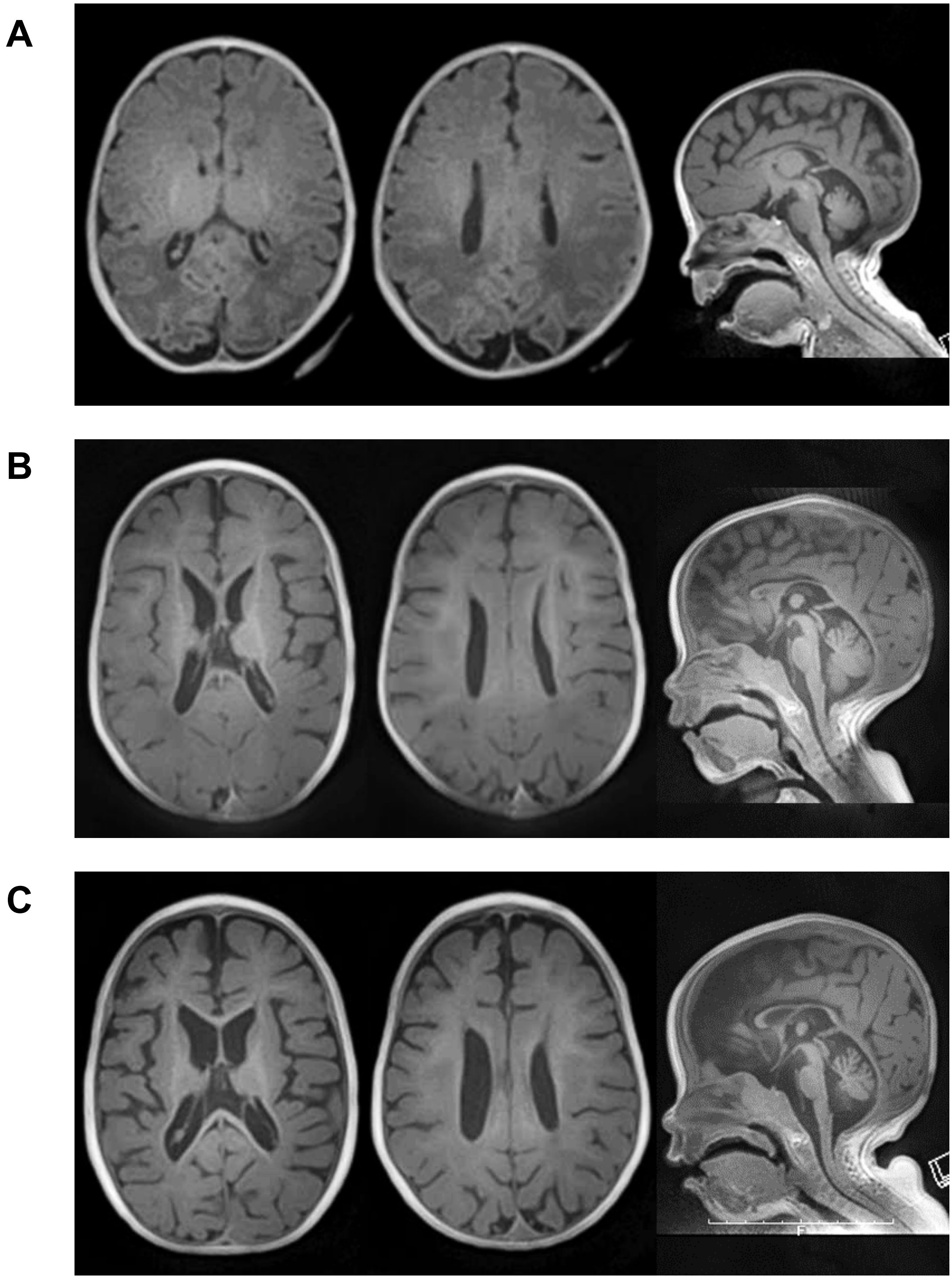

Figure S1A–C. Clinical findings of Patient 1 with a P563L variant in *NSF*. Brain MRI images obtained at 2 months (**A**), 8 months (**B**), and 1 year (**C**). Left and center panels are axial T1-weighted images, whereas the right panels are sagittal T1-weighted images (**A**–**C**). These images show progressive atrophy of cerebral cortex, brainstem and cerebellum, as well as delayed myelination.

D

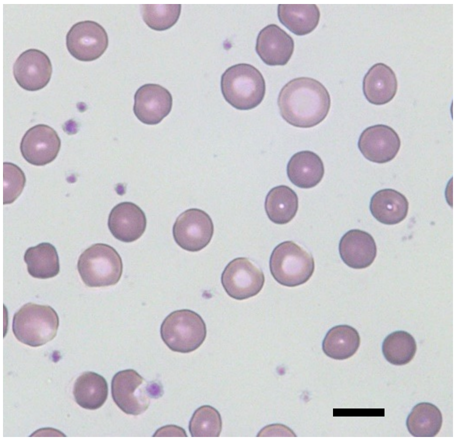

E

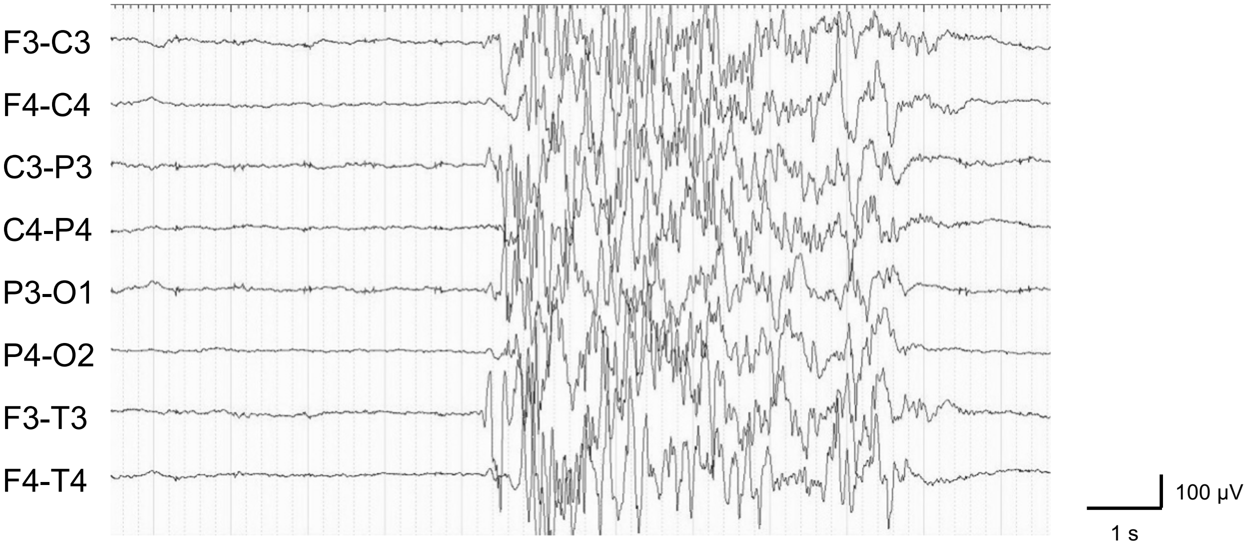

F

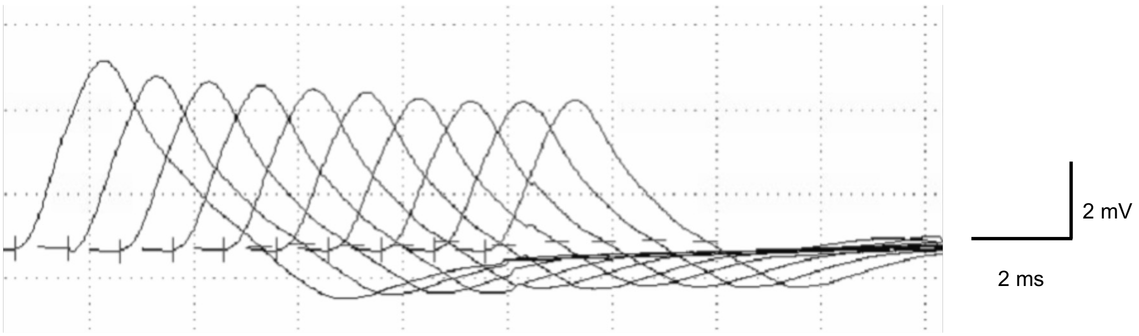

Figure S1D–F. Clinical findings of Patient 1 with a P563L variant in *NSF*. (**D**) A microscopic image of a peripheral blood smear at age 4 years. Anisocytosis of red blood cells is found. Scale bar = 10 µm. (**E**) Electroencephalography showing a burst-suppression pattern at age 3 years. (**F**) A 3-Hz repetitive stimulation test in the ulnar nerve showing a decremental pattern of contraction amplitudes in the abductor digiti minimi muscle at age 3 years.

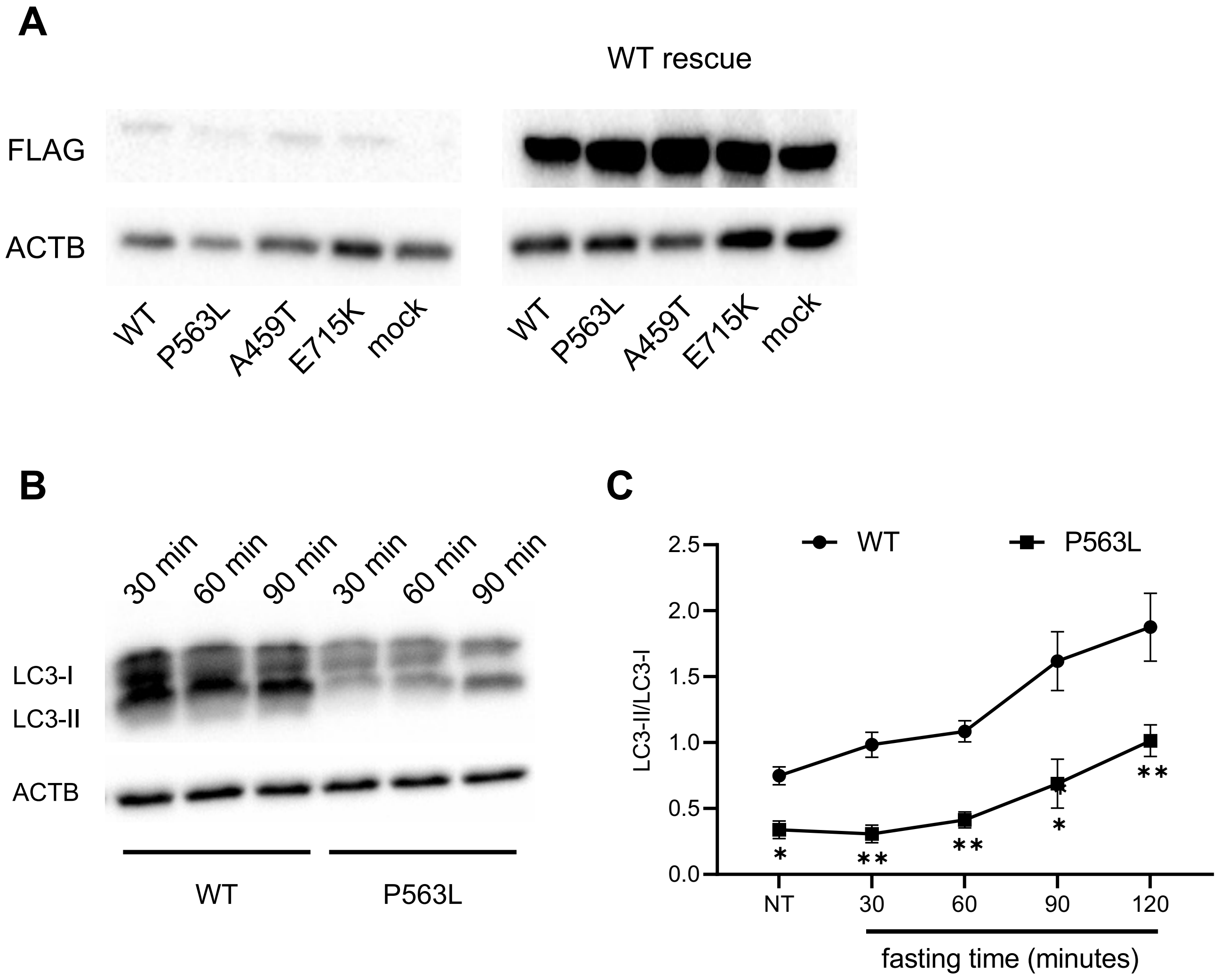

Figure S2. NSF^{WT} overexpression and time course of autophagic activation during fasting. (**A**) Representative images of Western blot analysis for FLAG in PC12 cells that stably express FLAG-tagged NSF. The left side shows cells without rescue treatment. The right side shows cells with rescue treatment by transient overexpression of NSF^{WT}. Images of both sides are acquired at the same exposure time. (**B**) A representative image of Western blot analysis for LC3 in PC12 cells that stably express NSF^{WT} or NSF^{P563L} in each time point. (**A** and **B**) ACTB was used as a loading control. (**C**) The time course of the ratio of LC3-II to LC3-I during fasting. A quantitative densitometric analysis of Western blots for LC3 in PC12 cell lines that stably express NSF^{WT} or NSF^{P563L} is performed to calculate the ratio. The data represent the mean ± SEM of three independent experiments. Statistical difference between WT and P563L is evaluated using Welch's t-test. *$p<0.05$, **$p<0.01$.

Abbreviation: NT, no treatment

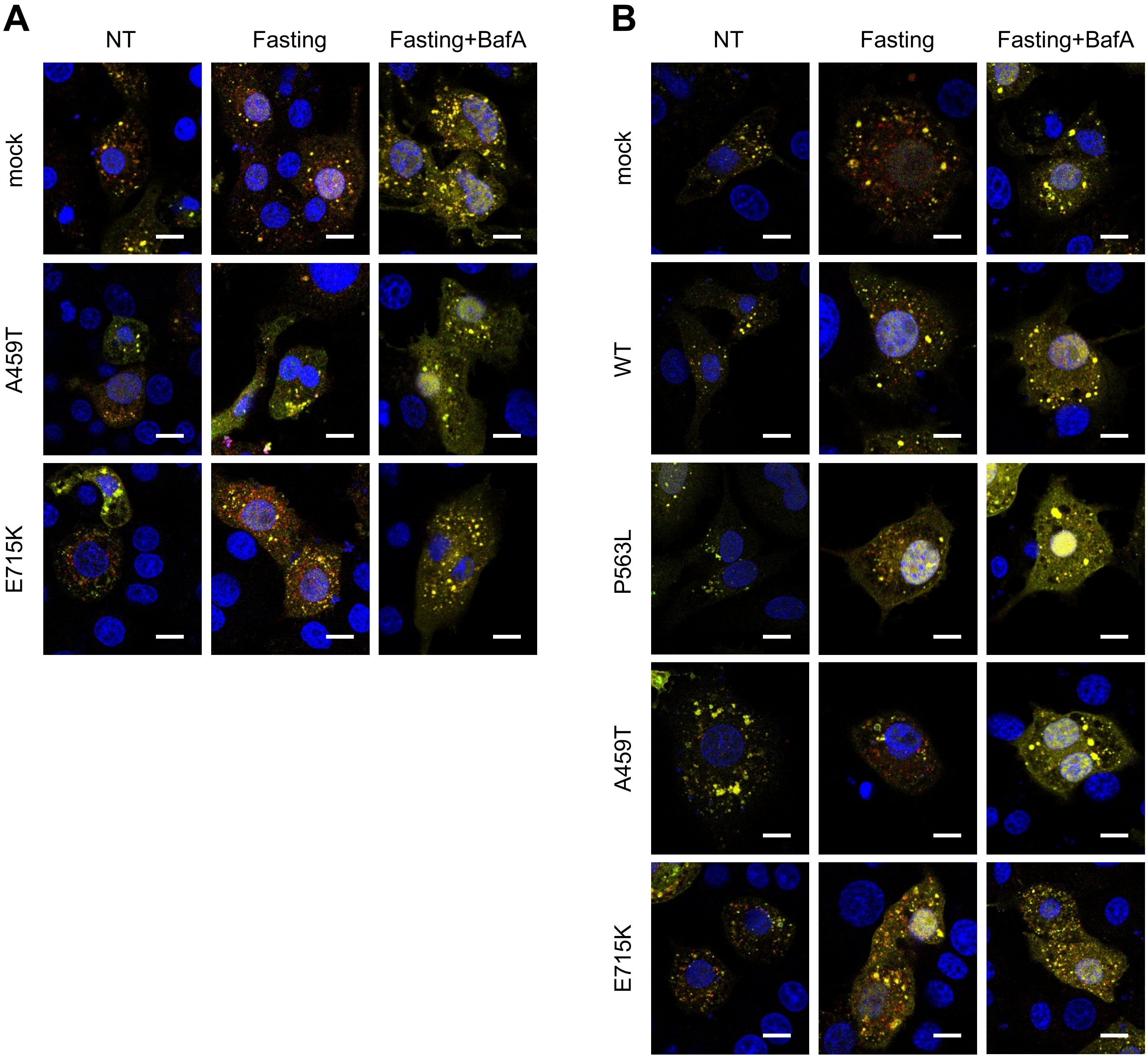

Figure S3. Representative confocal microscopic images of PC12 cell lines transfected with tandem-fluorescent LC3. (**A**) PC12 cells without NSF transfection (mock) and PC12 cells that stably express NSF^{A459T} or NSF^{E715K}. (**B**) Transient overexpression of NSF^{WT} normalized the autophagic response to fasting or BafA in PC12 cell lines that expressed NSF^{DEE}. (**A** and **B**) The yellow puncta indicate autophagosome, and the red puncta indicate autolysosome.

Abbreviation: NT, no treatment; BafA, bafilomycin A1. Scale bar = 10 μm

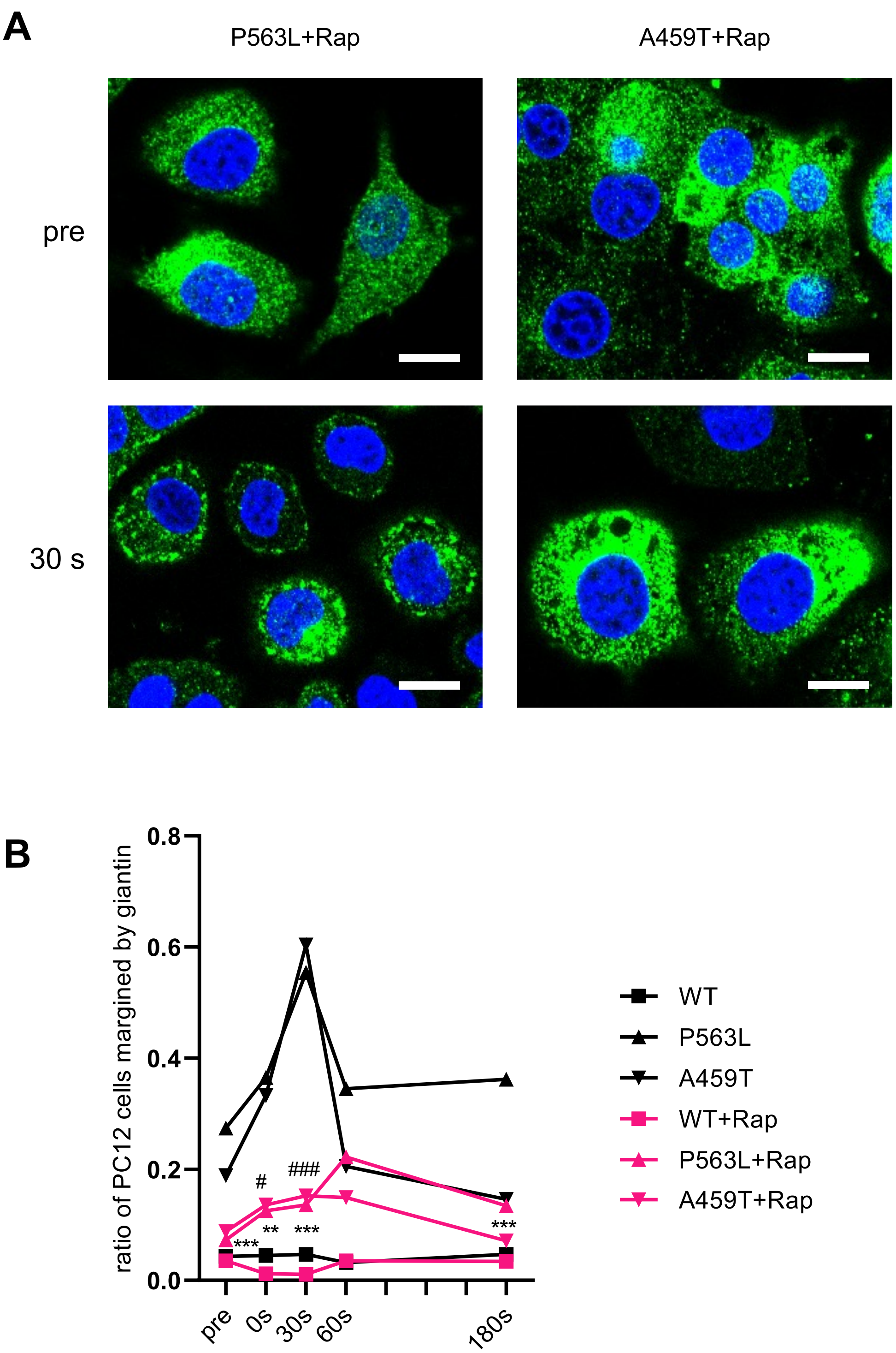

Figure S4. Rapamycin improved disturbed recycling of vesicular membrane protein in PC12 cells that stably express NSF^{DEE} . (**A**) Representative confocal microscopic images of immunofluorescence for giantin (green) in PC12 cell lines that stably express NSF^{P563L} or NSF^{A459T} cultured with 200 nM rapamycin (Rap). These images are obtained at baseline and 30 s after 80 mM KCl stimulation. The nuclei are stained with DAPI. Scale bar = 10 μm. (**B**) The ratio of giantin-margined cells in each PC12 cell lines. The data represent the mean ± SEM of 40-90 cells. Statistical difference is evaluated using chi-squared test and Fischer's exact test. $^{**}p<0.01$, $^{***}p<0.001$ P563L versus P563L+Rap, $^{\#}p<0.05$, $^{\#\#\#}p<0.001$ A459T versus A459T+Rap.

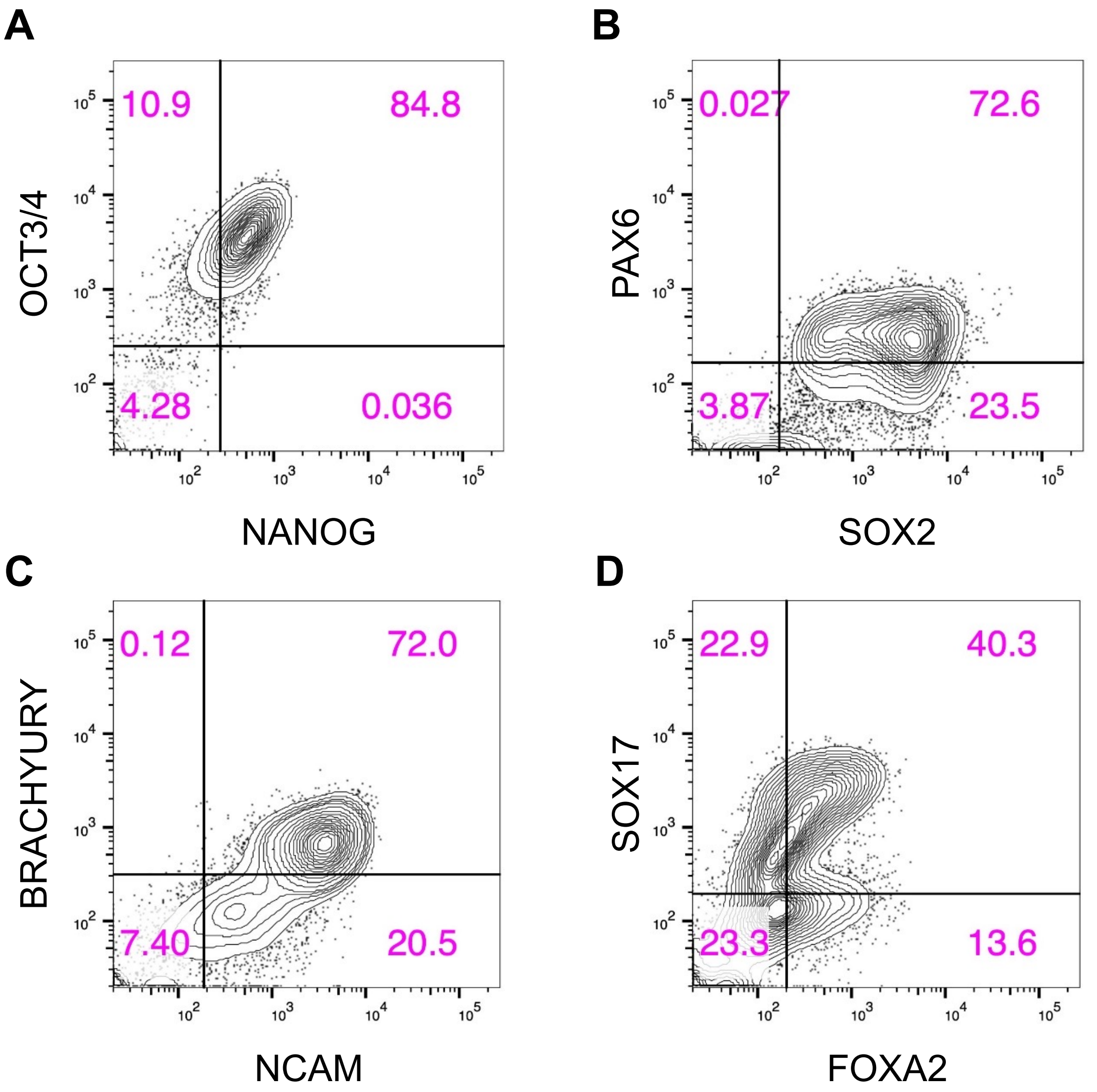

E

mRNA expression						Residual plasmid				Originated cell type		
OCT			NANOG			CAG		EBNA1		TRD	IGH	Judge-ment
Avg	RQ min	RQ max	Avg	RQ min	RQ max	copy /cell	SD	copy /cell	SD	allele #	allele #	
1.003	0.935	1.077	1.080	1.022	1.141	0.102	0.014	0.125	0.006	1.99	1.86	nonT/B

Figure S5. Characterization of iPS cells established from Patient 1 with a P563L variant in *NSF*. (**A**-**D**) Fluorescence-activated cell sorting-based analysis for expression of marker proteins in iPS cells. (**A**) Pluripotency markers (OCT3/4 and NANOG) in undifferentiated iPS cells. (**B**-**D**) Specific markers in iPS cells that are differentiated into each lineage. (**B**) Ectoderm markers (PAX6 and SOX2). (**C**) Mesoderm markers (BRACHYURY and NCAM). (**D**) Endoderm markers (SOX17 and FOXA2). Each number indicates the percentage of cells. (**E**) Summary of the results of OCT3/4 and NANOG mRNA expression, residual plasmid analysis, and originated cell type analysis. Details of these analyses are provided in Supplementary Methods.

Abbreviation: Avg, average; RQ, relative target quantity; SD, standard deviation; #, number

Table S1. Summary of the whole exome sequencing in Patients 1 and 2

Gene	Chromosome	gDNA (GRCh37)	cDNA		Amino acid
**Patient 1**					
De novo					
NSF	chr17	g.44791279C>T	NM_006178.3	c.1688C>T	p.Pro563Leu
C15orf43	chr15	g.45270792T>C	NM_152448.2	c.629T>C	p.Met210Thr
ABCA8	chr17	g.66924171C>A	NM_001288985.1	c.1159G>T	p.Ala387Ser
Compound heterozygous					
TTLL4	chr2	g.219603267G>A<br>g.219604866C>T	NM_014640.4	c.868G>A<br>c.1573C>T	p.Ala290Thr<br>p.Arg525Cys
ZNF131	chr5	g.43161380A>G<br>g.43175096A>T	NM_001297548.1	c.401A>G<br>c.1733A>T	p.Glu134Gly<br>p.Glu578Val
KRBA1	chr7	g.149425736G>C<br>g.149430426T>C	NM_001290187.1	c.1699G>C<br>c.2482T>C	p.Glu567Gln<br>p.Cys828Arg
VEGFB	chr11	g.64003311C>T<br>g.64004660C>T	NM_003377.4	c.130C>T<br>c.376C>T	p.Arg44Cys<br>p.Pro126Ser
PIEZO1	chr16	g.88788259delG<br>g.88798191T>G	NM_001142864.3	c.5171delG<br>c.3119A>C	p.Arg1724Serfs*8<br>p.Asn1040Thr
TYK2	chr19	g.10468591C>T<br>g.10476513G>A	NM_003331.4	c.2315G>A<br>c.691C>T	p.Arg772Gln<br>p.Arg231Trp
**Patient 2**					
De novo					
NSF	chr17	g.44782125G>A	NM_006178.3	c.1375G>A	p.Ala459Thr
SIPA1L2	chr1	g.232564237T>C	NM_020808.3	c.4330A>G	p.Thr1444Ala
PLXNA3	chrX	g.153689644C>T	NM_017514.4	c.800C>T	p.Ala267Val
Compound heterozygous					
ABCC10	chr6	g.43412898A>G<br>g.43415551G>A	NM_001198934.1	c.2876A>G<br>c.3835G>A	p.Asn959Ser<br>p.Val1279Met
Homozygous					
LAMA5	chr20	g.60903041G>A	NM_005560.4	c.4678C>T	p.Arg1560Cys

Table S2. Primary antibody information

Antibody	Provider	Catalog #	Application
NSF	Cell Signaling Technology	3924	Primary antibody
LC3	Sigma-Aldrich	L8918	Primary antibody
S6K	Cell Signaling Technology	2708	Primary antibody
pS6K	Cell Signaling Technology	9234	Primary antibody
Giantin	Biolegend	924301	Primary antibody
TUBB3	Biolegend	MMS-435P	Primary antibody
FLAG	Sigma-Aldrich	F1804	Primary antibody
ACTB	Wako	010-27841	Primary antibody
OCT3/4-Alexa647	BD Biosciences	560329	Primary antibody
NANOG-Alexa488	BD Biosciences	560791	Primary antibody
PAX6-Alexa488	BD Biosciences	561664	Primary antibody
SOX2-BV421	Biolegend	656114	Primary antibody
BRACHYURY-PE	R&D Systems	IC2085P	Primary antibody
NCAM-BV421	Biolegend	318328	Primary antibody
FOXA2-PE	BD Biosciences	561589	Primary antibody
SOX17-Alexa647	BD Biosciences	562594	Primary antibody
Mouse IgG1,κ-Alexa488	Biolegend	400129	Isotype control of Alexa488 for NANOG
Mouse IgG2a,κ-Alexa488	Biolegend	400233	Isotype control of Alexa488 for PAX6
Mouse IgG1,κ-PE	Biolegend	400112	Isotype control of FITC for FOXA2
Goat IgG-PE	R&D Systems	PIC108P	Isotype control of Alexa647 for OCT3/4 and SOX17
Mouse IgG1,κ-Alexa647	Biolegend	400130	Isotype control of Alexa647 for OCT3/4 and SOX17
Mouse IgG1,κ:-BV421	Biolegend	400158	Isotype control of BV421 for SOX2 and NCAM

Table S3. qRT-PCR primers used for iPS cell characterization

Gene	Taqman Assay ID	Probe label
hOCT3/4	Hs00999634_gH	FAM/MGB
hNANOG	Hs02387400_g1	FAM/MGB

Gene	Forward Primer	Reverse Primer	Probe Sequence	Probe label
GAPDH	TGCACCACCAACTGCTTAGC	TCTTCTGGGTGGCAGTGATG	ACTCATGACCACAGTCCA	VIC/MGB
cmCAG	GGCTCTGACTGACCGCGTTA	CAGAAAAGAAACAAGCCGTCATT	TGTAATTAGCGCTTGGTT	FAM/MGB
EBNA1	ATCAGGGCCAAGACATAGAGATG	GCCAATGCAACTTGGACGTT	TGTCCGGAGACCCCA	FAM/MGB
RNaseP1	CGGAGGGAAGCTCATCAGTG	CCCTAGTCTCAGACCTTCCCAA	TGCGTCCTGTCACTCCACTCCCA	VIC/TAMRA
TRD	AAAGGGAAAAAGGAAGAAGAGGGTTT	GGTCACGGCTGGGTGTTT	ACCTGTGTGGTAGGAAGGCACA	VIC/TAMRA
IGH	GAGTGACTGGCAGGGTTGAG	GGCCCCCAGCACCTTT	TCTCCCTTCCGCTGTTAGCC	FAM/NFQ-MGB

Supplementary Methods

OCT3/4 and NANOG mRNA expression analysis

mRNA expression of the pluripotent stem cell markers OCT3/4 and NANOG was confirmed by quantitative real-time PCR (qRT-PCR) with TaqMan™ assay using StepOnePlus™ Real-Time PCR Systems (Thermo Fisher). The primer and probe sequences are provided in Supplementary Table S2. The expression values of target genes were normalized by GAPDH expression from the same cDNA templates and calculated relative to those of the 201B7 iPSC line.

Abbreviation: iPSC, induced pluripotent stem cell

Residual plasmid analysis

The residual plasmids used for iPSC establishment were analyzed by TaqMan™ quantitative PCR using StepOnePlus™ Real-Time PCR Systems (Thermo Fisher). Primer and probe sequences of CMV and EBNA1 are designed on CAG-promoter region and cording region of EBNA1 gene (Supplementary Table S3). The numbers of residual plasmid were determined by a standard curve method with pCE-OCT3/4 episomal plasmid of known quantity using 50 ng genomic DNA of established iPSC at passage 4 to 6.

Originated cell type analysis

The originated cell type including T cell, B cell, or non-T/non-B cell lineage on established iPSC was analyzed by TaqMan™ quantitative PCR using StepOnePlus™ Real-Time PCR Systems (Thermo Fisher). Primer and probe sequences of TRD and IGH were designed on TRD, T cell receptor delta locus and IGH, and immunoglobulin heavy chain (IgH) locus, (Supplementary Table S3). Quantitative PCR was performed using TRD, IGH, and RNaseP1 primers and probes using 20 ng genomic DNA of established iPSC at passage 4 to 6. The number of TRD and IGH loci were analyzed using CopyCaller™ Software (Thermo Fisher) with the number of RNaseP1 loci as an internal standard. The cell type of origin was determined as T cell lineage if the number of TRD loci was 1 or 0, B cell lineage if the number of IGH loci was 1 or 0, and nonT/nonB cell lineage otherwise.

Validation of established iPSCs

Established (patient) iPSCs together with control human PSCs were differentiated into ectoderm, mesoderm, and endoderm lineages using the STEMdiff™ Trilineage Differentiation Kit (STEMCELL Technologies). hPSCs reaching 70–80% confluency were harvested with the TrypLE™ Select Enzyme (1X) (Thermo Fisher) and plated as a single-cell suspension in mTeSR1 medium (STEMCELL Technologies) containing 10 mM Y27632 (Wako) on 6-well plates coated with Matrigel (BD Biosciences). The cells were plated at 4.0×10^5, 2.0×10^5, and 4.0×10^5 cells per well for ectoderm, mesoderm, and endoderm differentiation culture, respectively, and differentiated following the manufacturer’s instructions.

For FACS-based evaluation of undifferentiated PSCs, each of the three germ layers (1.0 × 10^6 cells each) were fixed with 4% paraformaldehyde phosphate buffer solution (PBS) for 20 minutes at 4° C and washed twice with staining medium containing PBS with 2% fetal bovine serum. Samples were permeabilized with BD Perm/Wash buffer (BD Biosciences) for 15 minutes at room temperature and stained with fluorescence-conjugated primary antibodies listed in Supplementary Table S. The samples were washed with BD Perm/Wash buffer twice and suspended into the staining medium. Flow cytometric analysis was performed using LSR (BD Biosciences). FACS data were analyzed, and graphs were generated using FlowJo software (FlowJo LLC). For transcript-level assessment of differentiation capacity, qPCR was performed with a 384-well TaqMan™ hPSC Scorecard™ panel (Thermo Fisher) by QuantStudio™ 5 Real-Time PCR System (Thermo Fisher) using undifferentiated PSC and each of the three germ layer cDNA samples. Pluripotency and differentiation property into ectoderm, mesoderm, and endoderm lineages were scored using hPSC Scorecard Analysis software, which is available on the Thermo Fisher website (https://www.thermofisher.com/jp/en/home/life-science/stem-cell-research/taqman-hpsc-scorecard-panel.html).

Abbreviation: hPSC, human pluripotent stem cell
